# Integrative analysis of WDR12 as a potential prognostic and immunological biomarker in multiple human tumors

**DOI:** 10.3389/fgene.2022.1008502

**Published:** 2023-01-16

**Authors:** Refaat A. Eid, Muhammad Alaa Eldeen, Mohamed A. Soltan, Mubarak Al-Shraim, Majed Aldehri, Leena S. Alqahtani, Ghadi Alsharif, Sarah Albogami, Ibrahim Jafri, Eman Fayad, Moon Nyeo Park, Shabana Bibi, Mohammed Y. Behairy, Bonglee Kim, Mohamed Samir A. Zaki

**Affiliations:** ^1^ Pathology Department, College of Medicine, King Khalid University, Abha, Saudi Arabia; ^2^ Cell Biology, Histology & Genetics Division, Biology Department, Faculty of Science, Zagazig University, Zagazig, Egypt; ^3^ Department of Microbiology and Immunology, Faculty of Pharmacy, Sinai University, Ismailia, Egypt; ^4^ Anatomy Department, College of Medicine, King Khalid University, Abha, Saudi Arabia; ^5^ Department of Biochemistry, College of Science, University of Jeddah, Jeddah, Saudi Arabia; ^6^ College of Clinical Laboratory Sciences, King Saud bin Abdulaziz University for Health Sciences, Jeddah, Saudi Arabia; ^7^ Department of Biotechnology, College of Sciences, Taif University, Taif, Saudi Arabia; ^8^ Department of Pathology, College of Korean Medicine, Kyung Hee University, Seoul, South Korea; ^9^ Department of Biosciences, Shifa Tameer-e-Millat University, Islamabad, Pakistan; ^10^ Yunnan Herbal Laboratory, College of Ecology and Environmental Sciences, Yunnan University, Kunming, Yunnan, China; ^11^ Department of Microbiology and Immunology, Faculty of Pharmacy, University of Sadat City, Sadat City, Egypt; ^12^ Department of Histology and Cell Biology, College of Medicine, Zagazig University, Zagazig, Egypt

**Keywords:** WDR12, pan-cancer, prognosis, immunotherapy, expression

## Abstract

**Background**: Mammalian WD-repeat protein 12 (WDR12), a family member of proteins containing repeats of tryptophan-aspartic acid (WD), is a potential homolog of yeast Ytm1p and consists of seven repeats of WD.

**Aim of the study**: This study aims to investigate the potential oncogenic effects of WDR12 in various human malignancies throughout a pan-cancer analysis that has been carried out to examine the various patterns in which this gene is expressed and behaves in tumor tissues.

**Methods**: Herein, we used The Cancer Genome Atlas (TCGA) and various computational tools to explore expression profiles, prognostic relevance, genetic mutations, immune cell infiltration, as well as the functional characteristics of WDR12 in multiple human cancers.

**Results**: We found that WDR12 was inconsistently expressed in various cancers and that variations in WDR12 expression predicted survival consequences for cancer patients. Furthermore, we observed a significant correlation between WDR12 gene mutation levels and the prognosis of some tumors. Furthermore, significant correlations were found between WDR12 expression patterns and cancer-associated fibroblast (CAF) infiltration, myeloid-derived suppressor cells (MDSCs), tumor mutation burden, microsatellite instability and immunoregulators. Ultimately, pathway enrichment analysis revealed that WDR12-related pathways are involved in carcinogenesis.

**Conclusions**: The findings of our study are stisfactory, demonstrating that WDR12 could serve as a promising reliable prognostic biomarker, as well as a therapeutic target for novel cancer therapeutic approaches.

## 1 Introduction

Cancer is widely recognized as one of the most significant public health concerns due to its role as one of the prominent causes of mortality and morbidity (Ohn et al., 1997). Tumorigenesis is a complex process governed by the origin of cells, the location of the tumor, genetic alterations, and acquired and hereditary molecular changes (Rohrmoser et al., 2007). Even though several medications and therapies have been created to treat cancer, several issues, including medication side effects, drug resistance, high healthcare costs, and missed targets, are still present (Zhang et al., 2017). As a result, there is still an urgent need to research more reliable tumor biomarkers and spotlight the specific biochemical pathways implicated in the development of tumors.

Mammalian WD repeat protein 12 (WDR12), a member of the tryptophan-aspartic acid (WD) repeat-containing proteins, is a possible homolog of yeast Ytm1p and contains seven repeats of WD. WDR12 transcript is largely expressed throughout embryogenesis, with the highest possible level found in the thymus and testis of mouse models (Hölzel et al., 2005). WDR12 forms a stable complex with Pes1 and Bop1 called PeBoW, which is essential for the biogenesis of mammal ribosomes. As a component of PeBoW, WDR12 plays a vital role in processing the 32S precursor ribosomal RNA and cell growth (Hölzel et al., 2005; Rohrmoser et al., 2007). Furthermore, WDR12 is required for the formation of the 28S and 5.8S ribosomal RNAs, as well as the development of the 60S ribosomal subunit (Nagy et al., 2021). According to the findings, inhibiting the expression of WDR12 in neonatal myocytes led to a reduction in the activation of ERK1/2, p38 MAPK, and HSP27. This discovery might shed light on how WDR12 regulates cell proliferation and survival (Moilanen et al., 2015). However, with these sophisticated discoveries, the significance of WDR12 in other physiological systems is still largely a mystery (Lerch-Gaggl et al., 2002). It was found to be preferentially expressed in glioma stem-like cells (GSCs), a subset of tumor cells that initiate malignant growth and promote the therapeutic resistance of glioblastoma, compared to non-stem tumor cells and normal brain cells. Moreover, WDR12 depletion compromised GSC proliferation, inhibited GSC-derived orthotopic tumor growth, and extended animal survival (Mi et al., 2021). Another study that correlated glioblastoma with the overexpression of WDR12 demonstrated that the knockdown of WDR12 promoted apoptosis and inhibited the proliferation of tumor cells (Li et al., 2020a). Hepatocellular carcinoma was another type of tumor that experienced up-regulated expression of WDR12 where the knockdown of WDR12 expression resulted in reduced proliferation and migration of HepG2 and Huh-7 cells (Li et al., 2020b).

In recent years, various public cancer databases, such as The Cancer Genome Atlas (TCGA) and Gene Expression Omnibus (GEO), have been constructed as a result of the fast development of whole genome sequencing technology (Blum et al., 2018; Hutter and Zenklusen, 2018). We can now assess the clinical prognosis of genes of interest through a pan-cancer analysis thanks to the open access aspect of these publicly available databases. This allows us to compare and contrast the commonalities and differences among human malignancies (Cui et al., 2020). We utilized the TCGA project and the publicly accessible bioinformatics tools to implement a pan-cancer analysis of WDR12 to examine the roles and underlying mechanisms of WDR12 in the pathophysiology or clinical prognosis of malignant tumors. This included expression variations, correlations between expression profiles and survival, genetic modifications, immune cell infiltration, tumor mutation burden (TMB), microsatellite instability (MSI), immunoregulators, as well as relevant molecular pathways.

## 2 Materials and methods

### 2.1 WDR12 expression analysis

WDR12 expression profiles were examined throughout the “Gene DE” module of TIMER2 (tumor immune estimation resource, version 2) (http://time.cistrome.org/). For those without normal tissues in the TIMER2 database, we matched the TCGA and GTEx databases using the GEPIA2 (Gene Expression Profiling Interactive Analysis, version 2) web’s Expression Analysis-Box plots module (http://gepia2.cancer-pku.cn/#analysis). This allowed us to examine the differences in WDR12 expression between multiple malignancies and correlate with normal tissues (Tang et al., 2017). Furthermore, the ‘Pathological Stage Plot’ module of GEPIA2 was selected to examine the WDR12 expression profile at various pathological stages. Furthermore, the CPTAC module of UALCAN (http://ualcan.path.uab.edu/analysis.html) was used to calculate the levels of WDR12 protein expression in cancers and normal tissues that were directly analogous to these malignancies (Chandrashekar et al., 2017).

### 2.2 Immunohistochemistry staining

IHC images of WDR12 protein expression in normal tissues and seven cancerous tissues, including LUAD, BRCA, LIHC, Glioma, KICH, and COAD, were obtained from the HPA (Human Protein Atlas) (http://proteinatlas.org/) and analyzed to assess differences in WDR12 expressions.

### 2.3 Survival prognosis analysis

Initially, the GEPIA2.0 website was used to evaluate patient survivability, where WDR12 was tested in the “survival analysis” section; literally, the entire tumor listed in the TCGA cohort was chosen, and the heatmap was retrieved for the two possible server-side approaches was retrieved [the overall survival (OS) and disease-free survival (DFS)].

### 2.4 Gene alteration analysis

We were able to elucidate the genetic modification characteristics of WDR12 by using the “Cancer Types Summary” module that is available in the Web version of the cBioPortal (https://www.cbioportal.org) (Gao et al., 2013). The alteration frequency, the mutation type, and the Copy number alteration (CNA) of WDR12 in multiple human malignancies were retrieved after selecting “TCGA Pan-Cancer Atlas Studies” from the “Quick select” menu. This allowed the data to be more easily accessed. In particular, we utilized the ‘Comparison’ module of cBioPortal to identify the association between WDR12 genetic alteration and survival prognosis with or without WDR12 gene mutations. This was done to determine whether there was a significant difference between the two scenarios. The Mutations module highlighted the different protein structure domains due to genetic variations.

#### 2.4.1 The methylation analysis of WDR12

DNA methylation is a crucial element of the cellular epigenetic regulatory system, which governs gene expression profiles (Qu et al., 2013). The investigation of WDR12 methylation was conducted with the help of three separate databases. These databases were UALCAN (which can be accessed at http://ualcan.path.uab.edu/index.html), VShiny Methylation Analysis Resource Tool (SMART) (which can be accessed at http://www.bioinfozs.com/smartapp/), and MEXPRESS (which can be accessed at https://www.mexpress.be/) (Gao et al., 2013; Chandrashekar et al., 2017; Hutter and Zenklusen, 2018). We examined the levels of promoter methylation of WDR12 in both normal and cancerous tissues by using the “TCGA analysis” module included in UALCAN. On its website, SMART featured visual representations of DNA methylation locations, as well as CpG-aggregated methylation. Comparative analyses of promoter methylation levels in LGG and GBM were carried out with the help of the MEXPRESS database.

### 2.5 Immune cell infiltration analysis

We used the “Immune Gene” module of the TIMER2 Web server to investigate the relationship between WDR12 expression and immune infiltrates in every TCGA tumor. As immune cells, myeloid-derived suppressor cells (MDSCs) and cancer-associated fibroblasts (CAFs) were selected. Regarding measuring immunological infiltration, we used algorithms developed by TIMER, CIBERSORT, CIBERSORT-ABS, QUANTISEQ, XCELL, and MCPCOUNTER, and EPIC. Using the purity-adjusted Spearman rank correlation test, the *p*-values and partial correlation (cor) values were calculated. The findings were represented with a heat map and a scatter plot.

### 2.6 TMB and MSI analysis

TMB is a quantifiable immune-response biomarker identified by calculating the total number of gene coding errors, base substitutions, gene insertion, or deletion errors per million bases (Yarchoan et al., 2017). MSIs, short, tandemly repeated DNA sequences, are caused by DNA mismatch repair deficiency in cancer tissues. MSI with defective DNA mismatch repair is a clinically significant tumor biomarker (van Velzen et al., 2020) TMB and MSI scores were generated using somatic mutation data obtained from the TCGA database (https://tcga.xenahubs.net). Additionally, using Spearman’s method, we investigated whether there was a connection between the expression of WDR12, TMB, and MSI.

### 2.7 Correlation analysis between WDR12 and immunoregulators

An investigation of the relationship between WDR12 and immunomodulators, immunostimulators, immunoinhibitors, MHC molecules, TILs, receptors, and chemokines in various cancers was carried out with the help of the TISIDB portal (http://cis.hku.hk/TISIDB/).

### 2.8 Gene set enrichment analysis of WDR12

We utilized the STRING database to retrieve experimentally validated WDR12-binding proteins (https://string-db.org/). We specified the minimum desired interaction scoring rate to ‘Low confidence,’ the meaning of network edges to ‘evidence,’ the highest possible number of interactors to display to ‘not over than 50 interactors,’ and the active interaction sources to ‘experiments’. Furthermore, GEPIA2’s ‘Similar Gene’ module was used to identify the top 100 WDR12-correlated genes as well. As part of GEPIA2, correlation analysis was performed on WDR12 and these other genes. To retrieve heatmap data for the genes listed above, we employed TIMER2’s ‘Gene_Corr’ module. The WDR12 binding and interacting genes were analyzed using the Venn diagram viewer (http://bioinformatics.psb.ugent.be/webtools/Venn/).

WDR12 and related genes were analyzed for the GO and KEGG pathway enrichment using the DAVID database (https://david.ncifcrf.gov/home.jsp) (database for annotations, visualization, and integrated discoveries). Visualization and depiction of the pathways were accomplished using ggplot2 R packages.

### 2.9 Statistical analysis

The log2 transformation was used to normalize all gene expression profiles. Tumor samples and normal tissues were compared using a Wilcox test to determine the WDR12 expression differences. The KM curve was applied for the survival analysis of tumor patients. The partial Spearman method was used to investigate the relationship between two variables. To perform all statistical analyzes, we used R software (Version 4.0.2). Statistical significance was defined as *p* < 0.05.

## 3 Results

### 3.1 Upregulation of WDR12 in multiple human cancers

**FIGURE 1:**
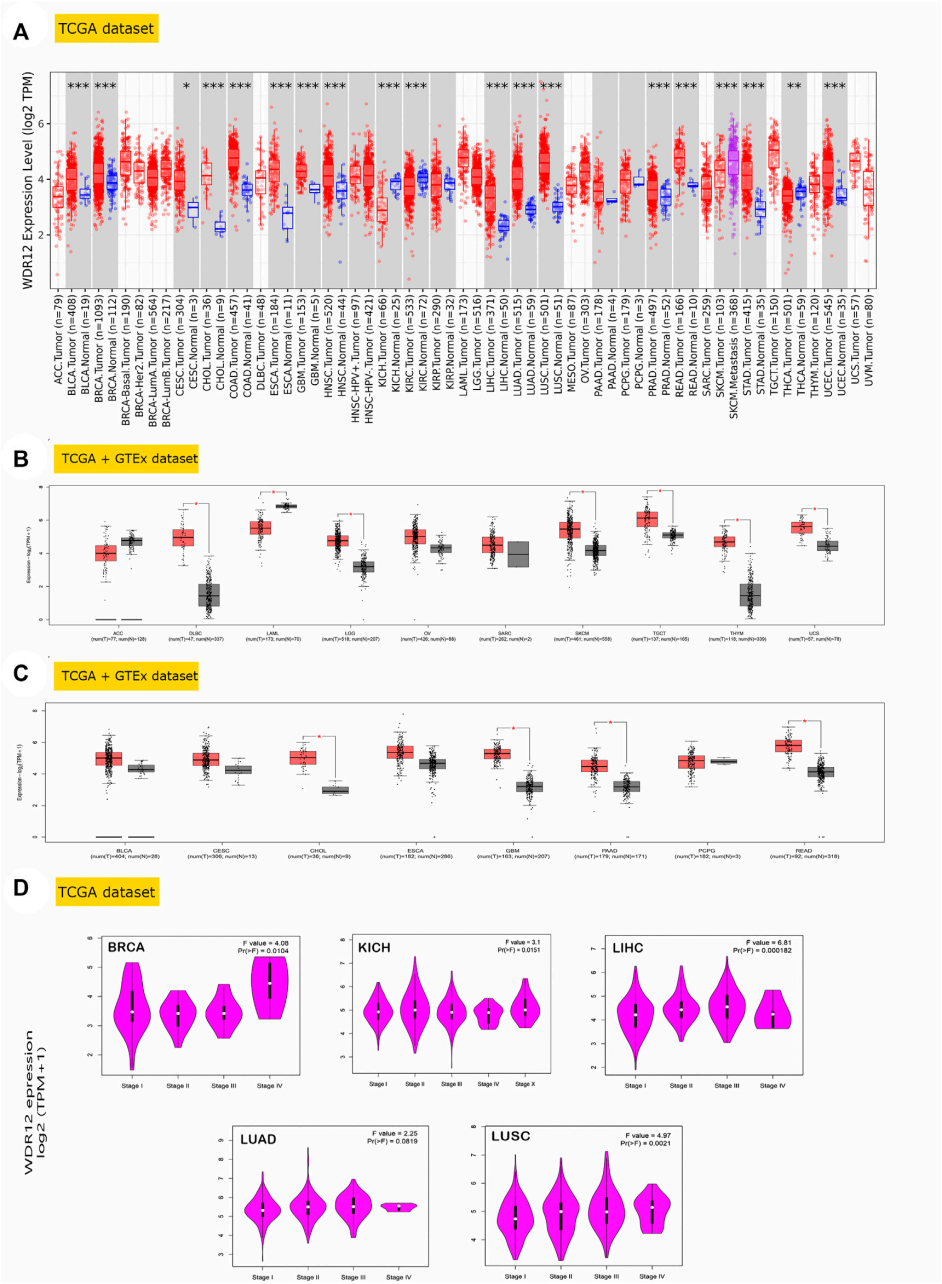
WDR12 expression assessment in human cancers. **(A)** Differential expression of WDR12 in a panel of TCGA tumors analyzed by TIMER2.0; **(B)** tumors that lack normal tissue for comparison in TIMER2.0. Database and experienced a trend of elevated WDR12 expression in tumor versus normal tissue when analyzed in the GEPIA database; **(C)** tumors that underwent a significant WDR12 protein expression versus normal tissue; **(D)** tumors experienced a positive correlation between WDR12 expression and the tumor stage.

As part of our investigation of the particular function of WDR12 in cancerous tumors, we compared the levels of WDR12 expression found in various sections of the TCGA database. Using the TIMER2 tool, we found that WDR12 was highly expressed in BLCA, BRCA, CESC, GBM, CHOL, COAD, ESCA, GBM, HNSC, LIHC, LUAD, LUSC, PRAD, READ, STAD, and UCEC (Figure 1A). Additionally, we used the GTEx dataset to further corroborate the expression profiles of WDR12 in a few more cancers that either did not have normal tissue matched in the TIMER2 dataset or had a small sample number (less than 20) of normal tissue that was used for expression comparison. From the tumor list that lacks a normal sample for comparison on the TIMER2 web server, DLBC, LGG, SKCM, TGCT, THYM, and UCS experienced a significant upregulation in WDR12 in comparison to normal tissue (Figure 1B). Moving to the tumors that had a small “normal tissue” sample size in the TIMER2 web server, all of the analyzed tumors showed a trend of WDR12 elevated expression in tumor *versus* normal samples, and this elevation was significant in tumors CHOL, GBM, READ, and PAAD (Figure 1C). It is worth noting that the sample size of “normal tissue” was less than 20 in 3 tumors, namely CESC, CHOL, and PCPG.

**FIGURE 2:**
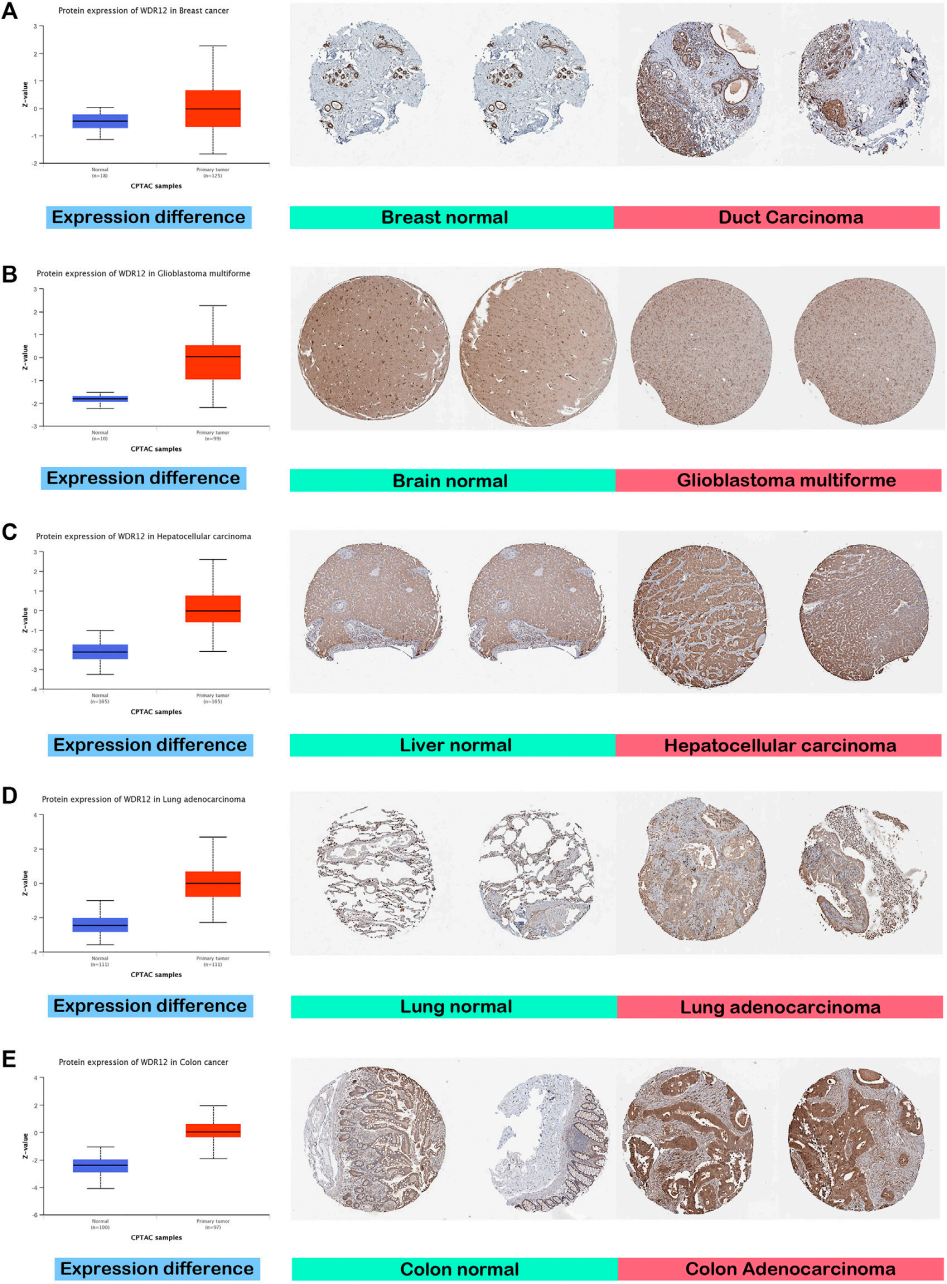
Tumors experienced a statistically significant higher WDR12 protein expression in the tumor sample versus the normal one (left side); IHC staining for normal tissue (middle) and cancerous one (left) demonstrated the same results. **(A)** Breast; **(B)** Brain; **(C)** Liver; **(D)** Lung; **(E)** Colon.

GEPIA2’s “Pathological Stage Plot” module investigated the relationship between WDR12 expression and tumor pathology stages. Statistically significant correlations were found in BRCA, KISH, LIHC, LUAD, and LUSC (Figure 1D). Moving to WDR12 protein level analysis, as shown in Figures 2A–E, WDR12 protein expression was significantly elevated in breast cancer, glioblastoma multiforme, hepatocellular carcinoma, LUAD, and colon cancer (*p* < 0.001). Moreover, we evaluated the IHC data provided by the HPA database and compared the findings with the differential protein expression. The results of the data analyses from these two databases were roughly equivalent. Normal lung, breast, brain, liver, and colon tissues were negative or moderately stained with WDR12 IHC, while cancerous tissues were moderately or strongly stained (Figures 2A–E).

### 3.2 WDR12 expression level exhibited a significant prognostic value

**FIGURE 3:**
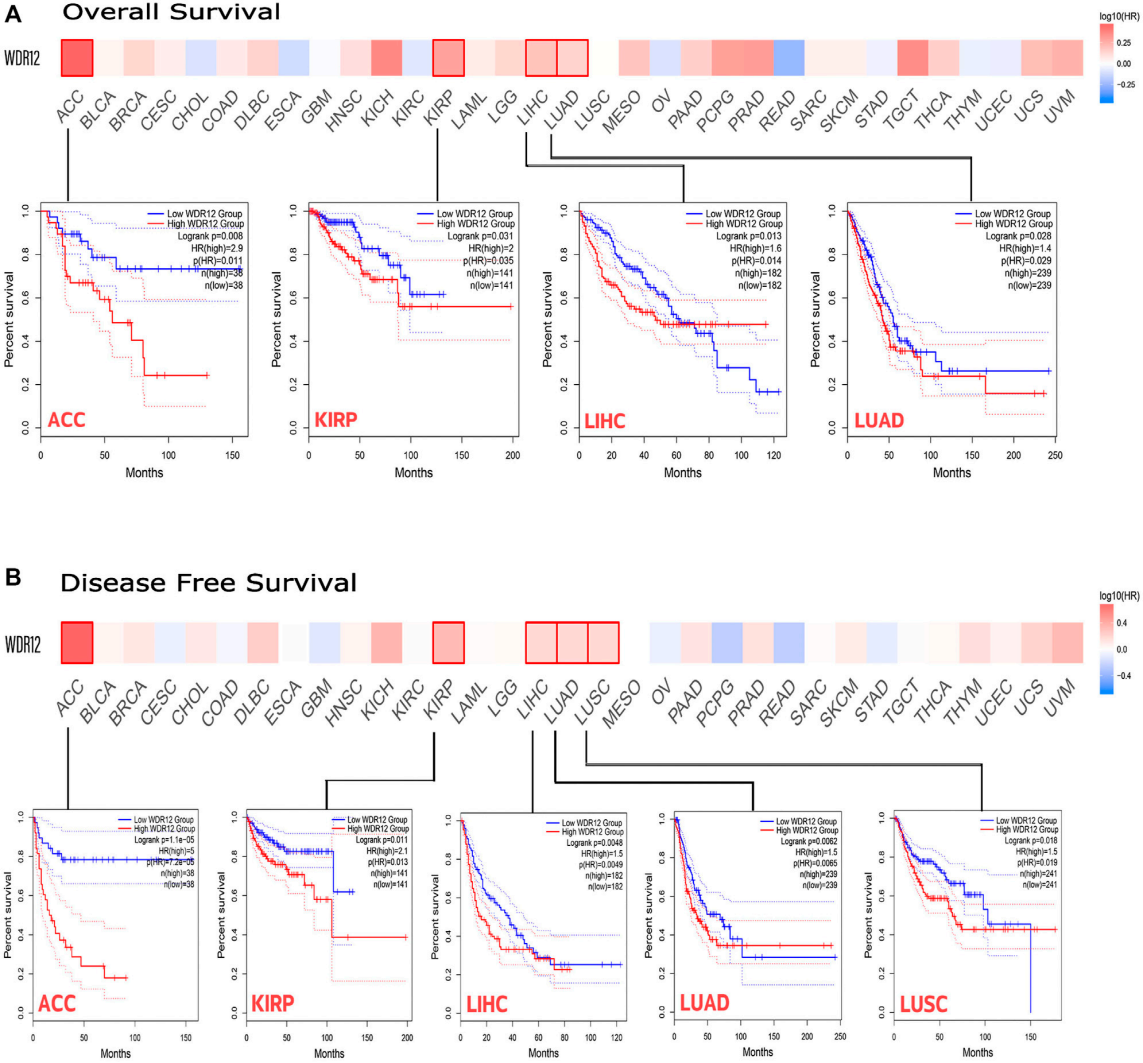
The correlation between WDR12 expression and the clinical outcome. **(A)** disease-free survival; **(B)** overall survival as assessed from the GEPIA database.

We used GEPIA2 web server to conduct a survival correlation study for each tumor. This was done to get a deeper insight into the relationship between WDR12 expression and the clinical outcome of cancer patients with various types of tumors. As shown in Figure 3A, those with high WDR12 expression levels were significantly linked to poor overall survival (OS) in ACC (*p* = 0.008), KIRP (*p* = 0.031), LIHC (*p* = 0.013), and LUAD (*p* = 0.028). However, high WDR12 expression was associated with longer OS in patients with ESCA (*p* = 0.2), KIRC (*p* = 0.23), READ (*p* = 0.24), and THYM (*p* = 0.86) (Fig. S1). In terms of DFS, highly expressed WDR12 was remarkably correlated with poor DFS in ACC (*p* = 1.1e-5), KIRP (*p* = 0.011), and LIHC (*p* = 0.0048), while downregulation of WDR12 has poor DFS in LUAD (*p* = 0.0062), and LUSC (*p* = 0.018) (Figure 3B).

### 3.3 The genetic mutation analysis of WDR12

**FIGURE 4:**
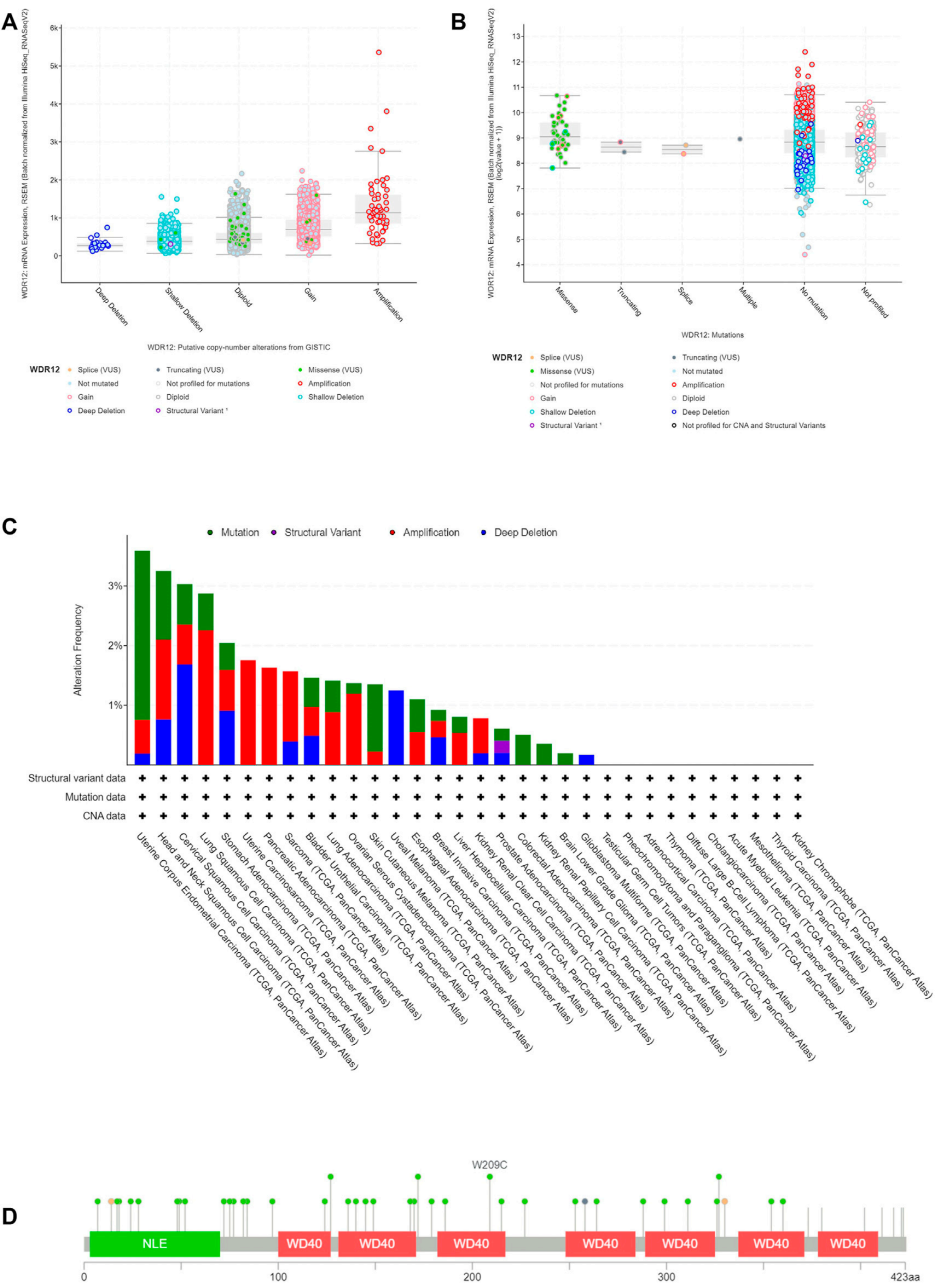
Mutation assessment for WDR12 using the cBioPortal tool. **(A)** Gene alteration analysis for WDR12; **(B)** mutation forms analysis for WDR12; **(C)** alteration frequency with mutation type in a panel of analyzed human cancers **(D)** map representation for sites and types of WDR12 mutations.

We analyzed WDR12 genetic alterations in multiple tumor samples from the TCGA database using the cBioPortal website. As illustrated in Figure 4A, the most prevalent CNA of WDR12 was amplification, whereas there were no deep deletions modifications. Additionally, missense mutations were predominantly seen in diploid and gain-type individuals. Analysis of mutation frequency revealed that missense mutations were the most common type of genetic modification (Figure 4B). Subsequently, we investigated WDR12 gene mutations and CNAs in human malignancies.

The results demonstrated that the most common type of WDR12 genetic modification is a gene mutation, which occurs most frequently in UCEC (∼1% frequency). The “amplification” type of CNA was the predominant type in LUSC, accounting for around 2% of all changes. WDR12 gene alterations are the most prevalent (3.5% frequency) in UCEC (Figure 4C).

Figure 4D shows the types, locations, and frequency of WDR12 genetic modifications. We discovered that missense mutations were more prevalent at the W209C site in the WD40 domain, which was the most modified site.

### 3.4 Methylation data analysis of WDR12 in human cancers

**FIGURE 5:**
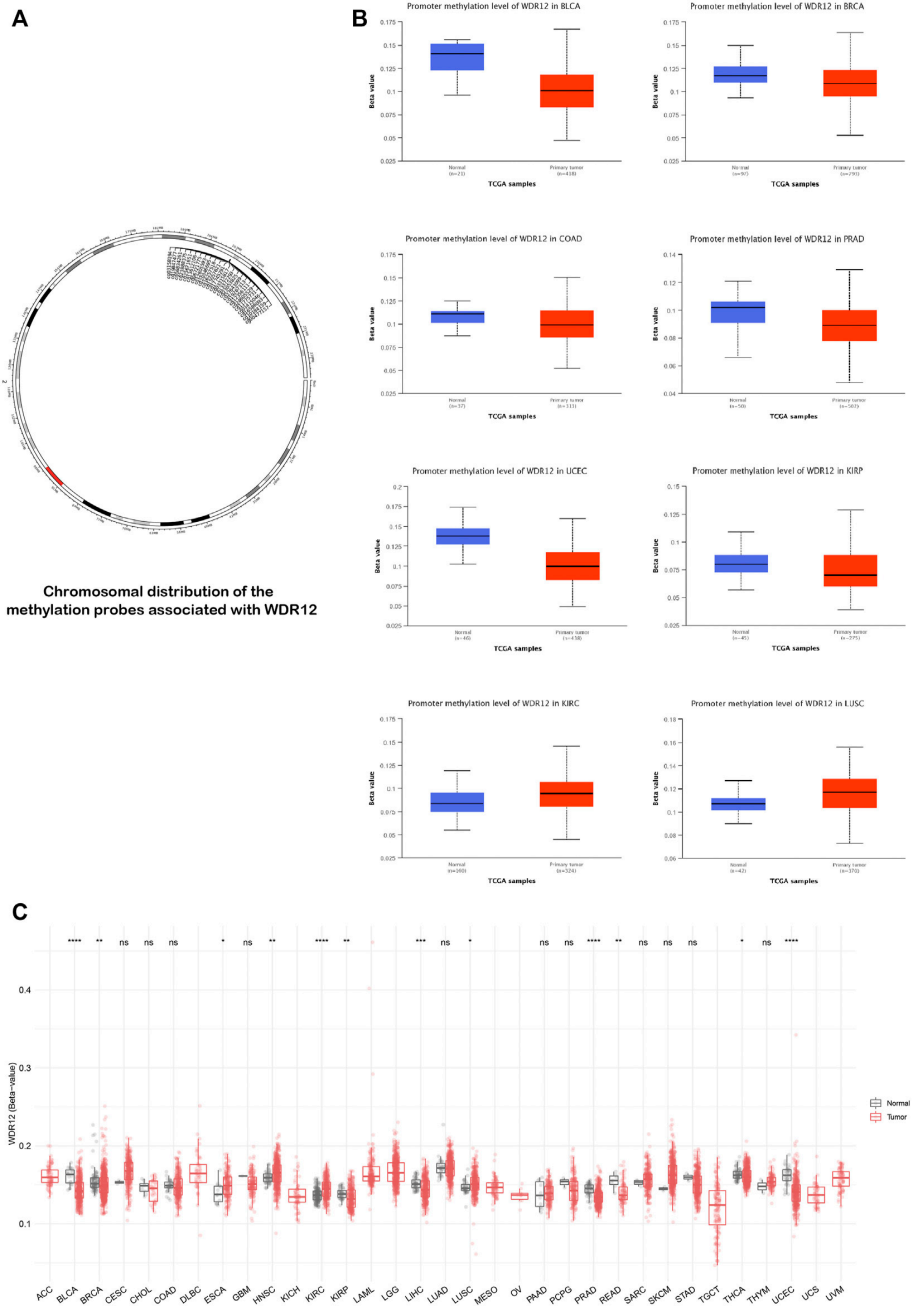
Differential methylation analysis of WDR12 in tumor samples versus normal ones. **(A)** Chromosomal distribution of methylation probes associated with WDR12; **(B)** tumors experienced higher methylation in the WDR12 promoter region in normal samples versus tumors as assessed by UALCAN analysis; **(C)** analysis of CpG-aggregated methylation of WDR12 in a list of human tumors.

The status of gene methylation has been extensively studied in several human cancers. Previous studies generally found that DNA hypermethylation was a major mechanism for silencing tumor suppressor genes (Anglim et al., 2008). On the other hand, oncogenes experienced a hypomethylation status as a mechanism for their activation to induce tumor progression (Romero-Garcia et al., 2020). We performed a methylation data analysis to examine the possible correlation between WDR12 DNA methylation and the pathophysiology of various cancers. Figure 5A illustrates the chromosomal distribution of WDR12-linked methylation probes as estimated by SMART APP, an interactive online application for visualization and analysis of DNA methylation. BLCA, BRCA, KIRP, LIHC, PRAD, READ, THCA, and UCEC tissues had a lower level of WDR12 methylation than normal tissues (Figure 5C). Using UALCAN online tools, we investigated WDR12 promoter methylation levels in different malignancies. In all tumor groups with at least 20 samples, the WDR12 promoter was less methylated in BLCA, BRCA, COAD, PRAD, KIRP, and UCEC than in normal tissues. By comparison, the degree of WDR12 promoter methylation was greater in LUSC and KIRC (Figure 5B). Most significant results favored WDR12 hypomethylation in the tumor sample *versus* normal. There was no substantial difference between CESC and HNSC promoter methylation levels (Supplementary Figure S2A). We also used the MEXPRESS approach to investigate the association between WDR12 methylation levels and tumor pathogenesis. Surprisingly, when the glioma grade increased, the promoter methylation levels of WDR12 reduced. As demonstrated in Supplementary Figure S2B, the histogram reflecting the level of WDR12 methylation in the promoter regions was considerably lower in GBM than in LGG.

### 3.5 Immune cell infiltration analysis of WDR12

**FIGURE 6:**
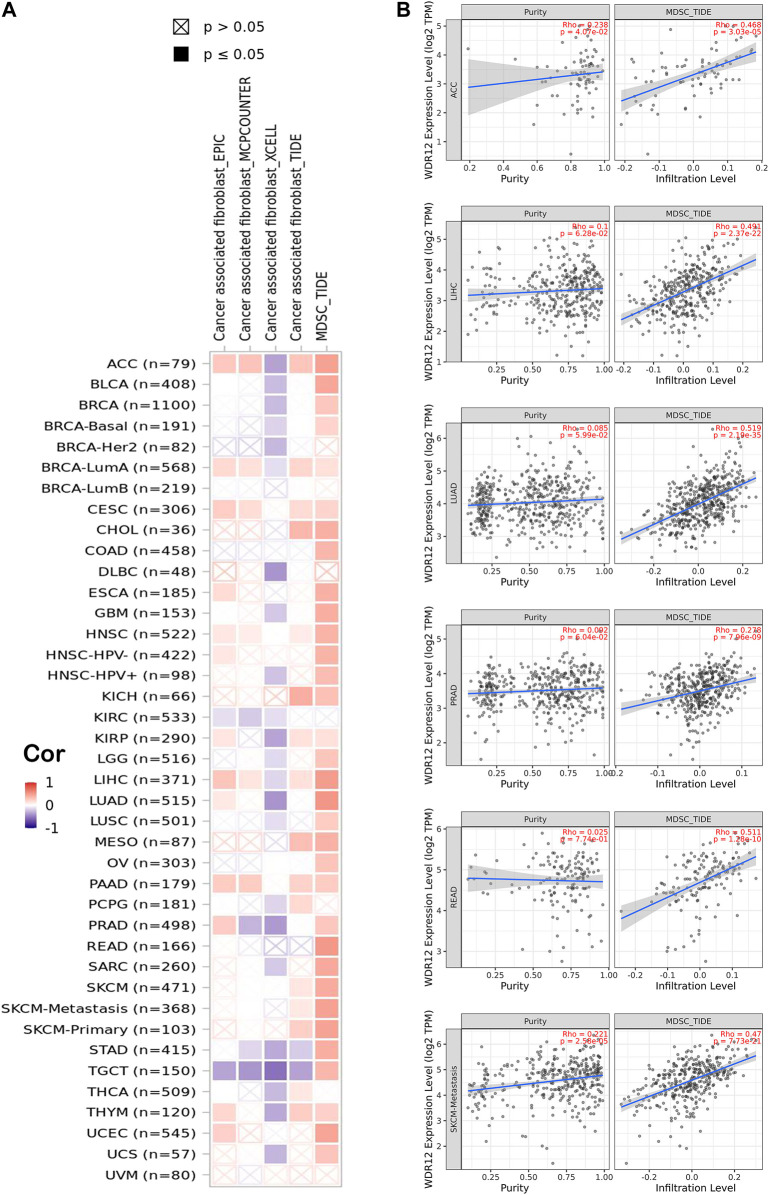
Correlation between WDR12 expression level and infiltration of **(A)** myeloid-derived suppressor cells (MDSC) and ;**(B)** Scatter plots demonstrate the correlation between the expression of WDR12 and the infiltration level of MDSC.

Previous studies have reported that the tumor microenvironment (TME) may play a crucial role in carcinogenesis and cancer cell immune evasion (Schumacher and Schreiber, 2015; Wu and Dai, 2017). Thus, TME significantly affects the treatment efficacy and overall prognosis of cancer. The TME is composed of tumor cells, immune cells, and stromal cells. Immune cells that enter the TME significantly affect tumorigenesis, progression, and metastasis (Anderson and Simon, 2020). CAFs and MDSCs have been shown to promote carcinogenesis in TMEs (Aizawa et al., 2019; de Cicco et al., 2020). Thus, we examined the relationship between WDR12 expression and tumor-infiltrating immune cells, such as CAFs and MDSCs. The TCGA database identified associations between WDR12 expression and MDSC and CAF infiltration levels. We identified significant positive correlations between the expression and MDSCs infiltration in all human cancers, excluding UVM, THCA, PCPG, KIRC, DLBC, BRCA-LumB, and BRCA-Her2. Several malignancies, including ACC, BRCA-LumA, CESC, CHOL, KISH, KIRP, LIHC, SKCM, and THYM, demonstrated significant positive correlations between the expression of WDR12 and the infiltration of CAFs (Figure 6A). Six tumors, including ACC, LIHC, LUAD, PRAD, READ, and SKCM, exhibited a significant positive correlation between WDR12 expression and MDSC and a negative correlation between the same gene expression and CAFs cell infiltration, as determined by filtration of the findings. Figure 6D shows scatter plots demonstrating the link between WDR12 expression and MDSCs infiltration level in these six tumors. MDSCs infiltration levels and WDR12 expression did not have statistically significant negative correlations; nevertheless, WDR12 expression and estimated CAFs infiltration values in STAD and TGCT did have statistically significant negative correlations (Supplementary Figure S3). Next, we investigated two additional types of immune cells but with established antitumor activity. The first cell type was NK T cells which experienced a negative correlation with the level of WDR12 and all tumors with significant results (Figure 6B). This finding confirmed the immunosuppressive role of WDR12 in tumor tissues. The second cell was CD8 which demonstrated a negative correlation with the level of WDR12 in the tumors HNSC, KIRC, KIRP, STAD, THCA, and UCS. It’s worth noting that few tumors, including BRCA and UVM, showed a positive correlation between the level of WDR12 and CD8 infiltration (Figure 6C).

### 3.6 Correlation analysis of WDR12 expression with TMB and MSI

**FIGURE 7:**
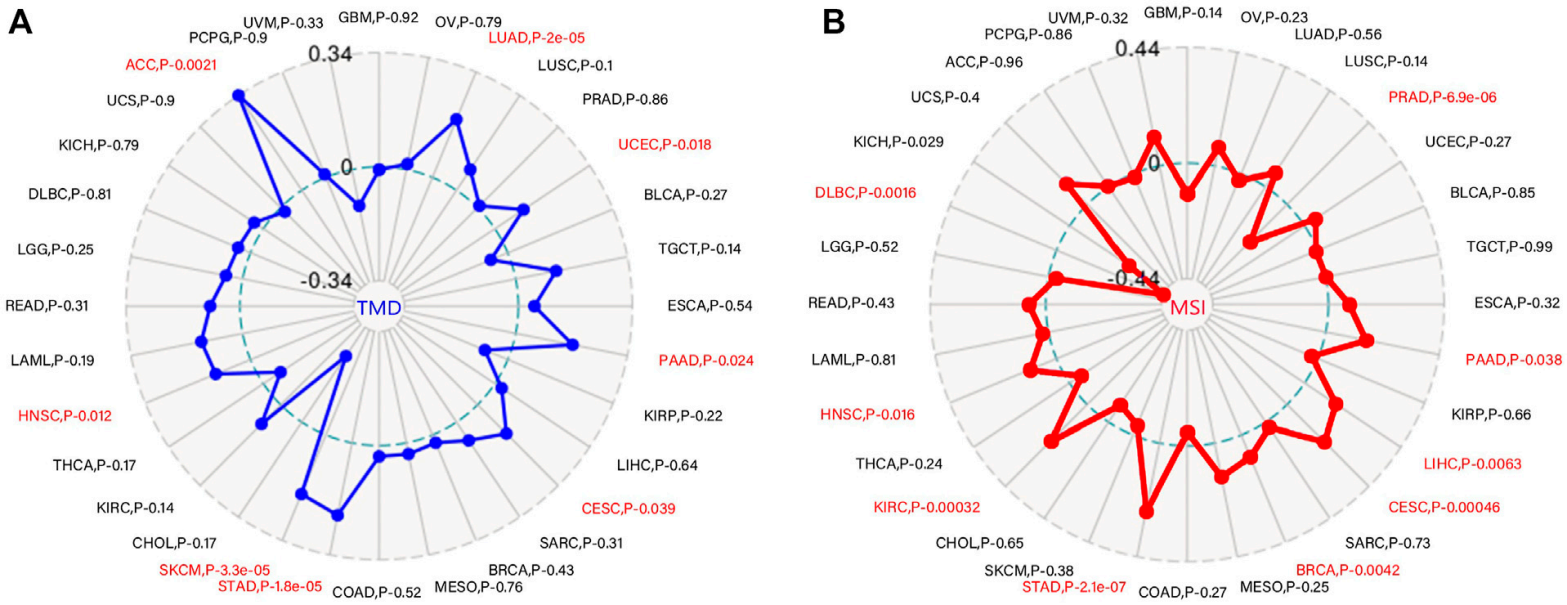
Correlations of WDR12 expression with MSI and TMB. **(A, B)** are radar charts showing the overlaps of WDR12 with TMB and MSI, respectively.

TMB is a promising therapeutic marker of the immunotherapy response. In addition, MSI acts as a biomarker for immune-checkpoint inhibitors, intimately linked to the advancement of the vast majority of malignancies (Fumet et al., 2020, Pino and Chung, 2011). MSIs in PRAD, LIHC, PAAD, CESC, BRCA, STAD, KIRC, KICH, and HNSC were positively correlated with WDR12 expression, while MSIs in DLBC were negatively correlated (Figure 7A). Our research results also revealed that WDR12 expression in LUAD, UCEC, PAAD, CESC, STAD, SKCM, HNSC, and ACC was significantly and positively correlated with TMB (Figure 7B).

### 3.7 Correlation analysis of WDR12 expression with immunoregulators

**FIGURE 8:**
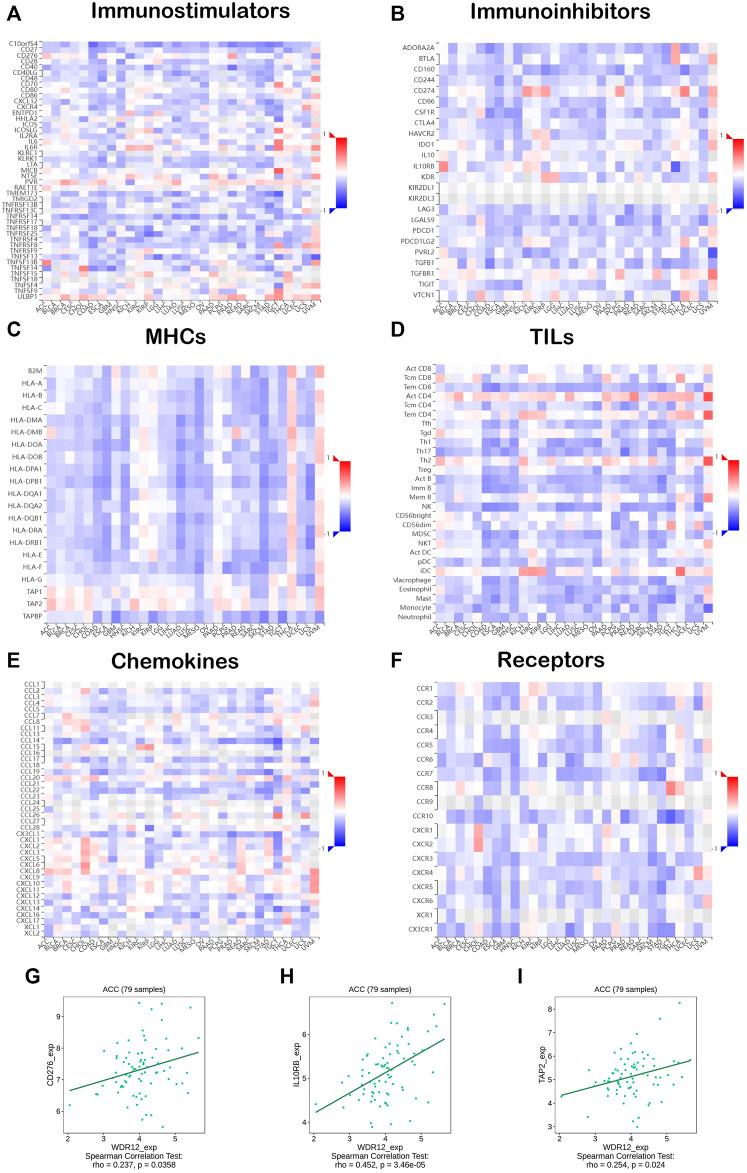
Correlation analysis of WDR12 expression with immunoregulators. Heatmaps correlating WDR12 with **(A)** Immunostimulators; **(B)** Immunoinhibitors; **(C)** MHCs; **(D)** TILs; **(E)** Chemokines; **(F)** Receptors. Plots **(G–I)** show the correlation between WDR12 and CD276, IL10RB, and TAP2, respectively.

The co-expression of WDR12, immune-related genes, and tumor-infiltrating lymphocytes (TILs) was investigated. All the immune-related genes studied encoded MHC, immunological activation, immunosuppression, chemokine, and chemokine receptor proteins. Our findings revealed that approximately all immune-related genes were significantly correlated with WDR12, and the majority were adversely associated with WDR12 in most malignancies (Figures 8A–F). To make a close investigation for the correlation between WDR12 and immune-related molecules, we selected ACC as a tumor model and molecules belonging to three classes, namely CD276 (immunostimulator), IL10RB (immunoinhibitor), and TAP2 (MHC-related molecule) where we found a significant correlation between these molecules and the expression level of WDR12 (Figures 8G–I). These results implicate that WDR12 can interfere with the tumor progression through its modulating effect on several classes of the immune-related molecule and consequently affects the immune response against a growing tumor. This characteristic has been explored in the last few years and resulted in the appearance of a novel line of cancer treatment, cancer immunotherapy (Finck et al., 2020).

### 3.8 Analysis of protein interactions and correlations with WDR12

Based on the results mentioned above, it is found that WDR12 has a clear association with cancer patients’ survival and affects immune cells in the TME. Consequently, it is important to analyze the potential molecular mechanisms of this gene in several tumors.

**FIGURE 9:**
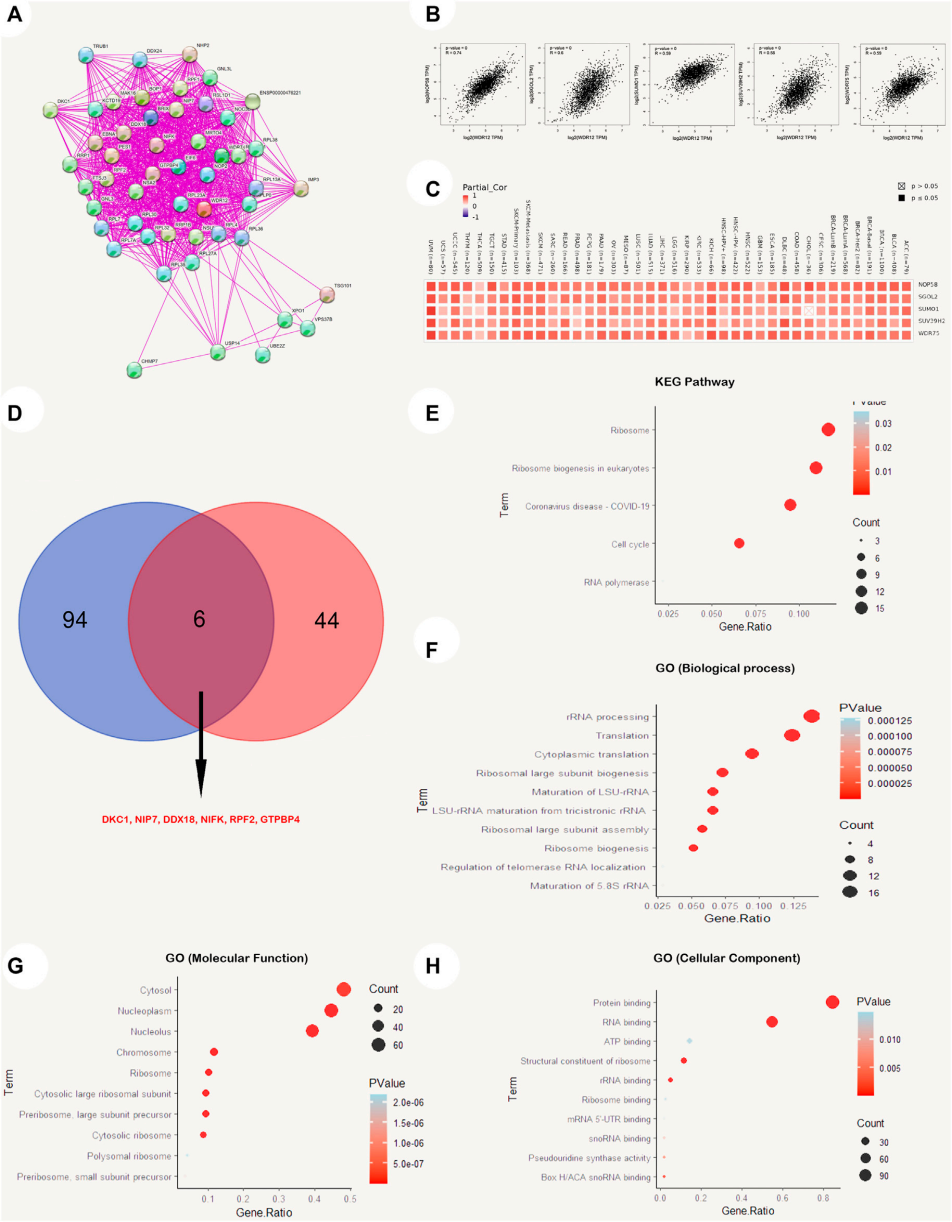
WDR12-protein network interactions. **(A)** A map of top 50 WDR12 interacting proteins as determined by STRING database; **(B)** expression correlation between WDR12 and genes (NOP58, SGOL2, SUMO1, SUV39H2 and WDR75) as determined by GEPIA2; **(C)** heatmap for top 5 WDR12-correlated proteins in the tumor tissue; **(D)** Venn diagram showing the intersection between WDR12 interacting and correlating proteins; **(E–H)** KEGG/GO enrichment analysis based on WDR12-binding and interacted genes.

As a result, we explored the possible underlying mechanisms of WDR12 in carcinogenesis by conforming to a network of fifty WDR12-binding protein interactions based on the STRING online database with supporting evidence from various experiments (Figure 9A). We then extracted the top 100 genes most strongly associated with WDR12 expression using GEPIA2. According to Figure 9B, WDR12 expression was positively correlated with NOP58 (R = 0.74), SGOL2 (R = 0.6), SUMO1 (R = 0.59), SUV39H2 (R = 0.58), and WDR75 (R = 0.59) (all *p* < 0.001). Furthermore, a heatmap generated through the ‘Gene_Corr’ module at TIMER confirmed the significant positive correlation between these five genes and WDR12 in the complete list of TCGA cancers (except CHOL, where the correlation with SUMO1 was insignificant) (Figure 9C). Furthermore, we performed an intersection analysis of the 50 WDR12 binding proteins and the top 100 correlated genes and found DKC1, NIP7, DDX18, NIFK, RPF2, and GTPBP4 were the common members through a Venn diagram (Figure 9D).

A new dataset was generated from the combination of two lists (WDR12 interacted genes from the STRING database and WDR12 associated genes from the GEPIA2 database), where this new list was subjected to enrichment analysis. Firstly, the KEGG pathway analysis reveals that “cell cycle” as well as “ribosome biogenesis” could be the potential methods by which WDR12 promotes carcinogenesis (Figure 9E). Secondly, GO enrichment analysis suggests that genes that directly interact with or were related to WDR12 were mainly correlated with the biological mechanisms of “rRNA processing”, “cytoplasmic translation,” and “ribosomal large subunit biogenesis” (Figure 9F), with the molecular functions of “protein binding” and RNA binding” (Figure 9G), and with the cellular components of the “cytosol”, “nucleoplasm” and the “nucleolus” (Figure 9H). It is important to mention that the cutoff *p*-value for the significant results in the enrichment analysis was *p* < 0.05, where only the top significant results (with the lowest *p*-value) were visualized using R packages.

## 4 Discussion

Cancer is a serious public health problem, and its incidence continues to increase annually. Although standard surgical resection, radiation, and chemotherapy have been used successfully as treatment options, several cases failed to get a positive response with these treatment options and still suffering (Fernández, 2014). This complicated tumor status underscores the need for a comprehensive strategy that studies a specific gene with tumorigenesis at several points of analysis, an approach that might provide us with novel treatment options. WDR12 has been reported in several previous studies to be associated with cancer progression in several models of human tumors. In colorectal cancer, WDR12 was found to be associated with tumor progression by promoting proliferation and inhibiting apoptosis where *in-vivo* tumor formation experiments in nude mice revealed that tumor area, volume, and weight were significantly lower for knockdown WDR12 (Fernández, 2014). In addition to that, whole-genome gene expression profiling revealed WDR12 as an implicated gene in colorectal cancer (Sun and Qian, 2018). Moreover, WDR12 was correlated with tumor progression in other human cancers including hepatocellular carcinoma (Li et al., 2020b) and glioblastoma (Li et al., 2020a), and was studied as a target for natural anticancer products in the breast cancer model (Lewinska et al., 2017). To this end, the present work employed a pan-cancer analysis to determine the oncogenic behavioral patterns of WDR12. We began our investigation by studying the distribution of WDR12 in human tissue, which was discovered to be expressed in various organs. An essential hallmark of oncogenic proteins is their overexpression in tumor tissue compared to normal tissue; as a result, we evaluated the expression of WDR12 in various malignancies using the TCGA, GTEx, and CPTAC databases. As described in the results section, WDR12 expression was significantly higher in several tumors, such as DLBC, THYM, GBM, LUAD, and STAD, than in equivalent normal tissues.

Survival analysis is a fundamental method for assessing the development of a disease and the responsiveness of a patient to therapeutic interventions (Nagy et al., 2021). Increased WDR12 expression levels were strongly associated with poor OS in ACC, KIRP, LIHC, and LUAD; and poor DFS in ACC, KIRP, and LIHC. Additionally, WDR12 genetic modification levels were significantly correlated with the prognosis of several malignancies. It is worth mentioning that some tumors, including ESCA, KIRC, READ, and THYM, experienced a better OS with elevated levels of WDR12, a point that requires further investigation to study the role and mechanism of WDR12 in these tumors and shows the importance of the pan-cancer studies that can reveal these opposite effects of the same gene in various types of human cancers. Genetic mutations are universally acknowledged to play a significant role in carcinogenesis (Anandakrishnani et al., 2019). Numerous gene mutations were discovered to be useful prognostic indicators for tumorigenesis; for example, genetic mutations in BRAF, PIK3CA, TP53, KRAS, and NRAS were discovered to be correlated with extramural venous invasion in baseline MRI, as well as poor clinical tumor regression and a trend towards much worse progression-free survival at 5 years (Sclafani et al., 2020). Earlier studies indicated that WDR12 polymorphisms were linked to genetic, developmental disorders, and malignancies (Yin et al., 2018).

Furthermore, Li et al. (Li et al., 2020b) verified the association between somatic WDR12 mutations and several inherited severe diseases. The cBioPortal tool was used to evaluate the altered patterns of WDR12 in a variety of human tumors, and the findings revealed that WDR12 was mutated in most tumors. WDR12 missense mutations were the most prevalent DNA mutations. Patients with LUSC with WDR12 genetic mutations had a worse prognosis for survival, which was later discovered. According to the data, patients with modified WDR12 were less likely to survive than those with unaltered WDR12. This suggests that WDR12 may be a useful predictor of patient survival and treatment responsiveness.

In numerous human malignancies, the state of gene methylation has been widely examined. According to earlier research findings, DNA hypermethylation is an important mechanism for tumor suppressor gene inhibition (Son et al., 2011; Xiao et al., 2014). However, on the other hand, hypomethylation served as a mechanism for oncogene stimulation to promote tumorigenesis (Medina-Aguilar et al., 2016). Next, we conducted a methylation analysis for WDR12. As expected, multiple cancers, namely BLCA, BRCA, KIRP, LIHC, PRAD, READ, THCA, and UCEC, exhibited hypomethylation in cancer tissues compared to normal samples. Furthermore, CpG aggregated methylation data showed that all significant findings favored hypomethylation of WDR12 in cancer tissues compared to normal samples (except for CHOL).

Immunotherapy has altered the landscape of cancer therapy by introducing novel and effective therapeutic approaches for numerous solid and hematologic cancers. While immunotherapy is not a new oncology branch, it has rapidly become one of the most promising in medicine (Khan et al., 2021). Improvements in checkpoint inhibitors, monoclonal antibodies, CAR-T cells, and anticancer vaccinations are among other significant breakthroughs. Subsequent research in immunotherapy, including the gut microbiota, in combination with checkpoint inhibitors and gene sequencing, continues to personalize therapies for cancer patients, offering limitless potential opportunities (Madden, 2018). For this reason, it was essential to examine the correlation between enhanced WDR12 expression in cancerous tissues and the infiltration of various immune cells into the tumors. It was discovered that MDSC positively influences tumor cell survival and metastasis (Ye et al., 2010; Condamine et al., 2015). In addition, it suppresses other tumor-fighting cells (CD8 T cells and NK cells), promotes angiogenesis, and participates in the development of cancer stem cells 40. Therefore, it was unsurprising that a high amount of MDSC infiltration was associated with a poor prognosis among cancer patients (Yin et al., 2020). In all tumor types investigated except for UVM, THCA, PCPG, KIRC, DLBC, BRCA-LumB, and BRCA-Her2, the current investigation revealed statistically significant and positively correlated associations between WDR12 expression and MDSCs infiltration. CAF is the second cell examined for its connection with WDR12 overexpression. This type of cell has a significant role in the fight against cancers in the early stages, participating in tumor immune surveillance and secreting several effector molecules (Shah et al., 2022).

Consequently, CAFs are a meaningful and informative supplement to current pathologic investigation and help to determine prognosis and therapeutic interventions. Identifying the processes by which CAFs and cancer cells interact reciprocally is essential for improving TME-focused cancer treatments (Knops et al., 2020). Various malignancies, particularly ACC, BRCA-LumA, CESC, CHOL, KISH, KIRP, LIHC, SKCM, and THYM, exhibited a significant positive correlation between the expression of WDR12 and the invasion of CAFs. In addition, we found that WDR12 was inversely correlated with immunostimulators, immunoinhibitors, MHCs, TILs, chemokines, and receptors in most malignancies, suggesting that the TME is complex in nature. In conclusion, these findings demonstrate that aberrant WDR12 expression plays a crucial role in TME. Combining WDR12 expression data, MDSC infiltration, and CAF infiltration, we may conclude that upregulation of WDR12 may serve as a biomarker for an inadequate immune response against a developing tumor.

As potential prognostic biomarkers for the effectiveness of immunotherapies in anticancer therapeutic interventions, TMB and MSI can offer additional insight into tumor behaviors. For this reason, we examined the correlation between WDR12 expression and TMB and MSI in all TCGA tumors. In LUAD, UCEC, PAAD, CESC, STAD, SKCM, and HNSC, a positive correlation was detected between WDR12 expression and TMB was detected. WDR12 expression was positively correlated with MSI in PRAD, LIHC, PAAD, CESC, BRCA, STAD, KIRC, HNSC, and KICH; however, it was negatively correlated with MSI in DLBC. The findings demonstrate that the WDR12 expression has a significant impact on the TMB and MSI, as well as on the immune checkpoint suppressive therapeutic response of patients. Our conclusions in this research suggest a research concern regarding the likelihood of using WDR12 expression in the tumor mentioned above as a possible biomarker for patients’ immunotherapy responsiveness.

Finally, we investigated the functional characteristics of differentially regulated WDR12 by including WDR12-binding proteins in addition to WDR12 expression-related genes in all TCGA cancers, followed by KEGG pathway enrichment and GO enrichment analyses. We retrieved the top 50 interacting proteins and the top 100 linked proteins to WDR12 in tumor tissue from the STRING and GEPIA2 databases, respectively, and found that DKC1 is a promising candidate. Among the top 100 genes with roughly similar expression patterns to WDR12 in TCGA tumors, the expression of WDR12 was significantly and positively correlated with the expression of NOP58, SGOL2, SUMO1, SUV39H2, and WDR75. WDR12 does not appear to interact physically with any of these five genes, even though they all play a role in the “cell cycle” and DNA replication. NOP58 is a possible prognostic biomarker for HCC because its upregulation is inversely correlated with OS in HCC patients (Wang et al., 2021). The correlation between high expression of SGOL2 and aggressive development and a poor prognosis in adrenocortical carcinoma is notable (Tian et al., 2020). NSCLC proliferation, colony formation, invasion, and NFB expression were increased by overexpression of SUMO1 (Ke et al., 2019). SUV39H2 maintains standard biological function, the loss of which promotes carcinogenesis, and its upregulation as an oncogene often leads to cancer initiation and progression (Li et al., 2019) WDR75 knockdown triggered the RPL5/RPL11-dependent stabilization checkpoint for p53, resulting in decreased cell proliferation and senescence (Moudry et al., 2022).

## 5 Conclusion

WDR12 is an oncogene that was shown to be overexpressed as mRNA and protein in many human malignancies. Its overexpression was found to be correlated with a poor prognosis. It influences the infiltration of multiple immune cells, with immunosuppressive cells MDSCs and CAFs infiltration associated favorably with WDR12 expression. Furthermore, it was discovered that TMB, MSI, and immunoregulators are associated with WDR12 expression in a subset of human cancers; therefore, these results potentially propose WDR12 as a prognostic biomarker of patients’ responsiveness to immunotherapy and a possible cancer treatment target.

Eventually, the present research work offers a trustworthy reference for the entire characteristics and roles of WDR12 in carcinogenesis, which can assist patients in selecting more precise immunotherapy regimens.

## Data Availability

The datasets presented in this study can be found in online repositories. The names of the repository/repositories and accession number(s) can be found in the article/Supplementary Material.
