# Supplementary figures and images for: Integrative analysis of WDR12 as a potential prognostic and immunological biomarker in multiple human tumors
Source: Front Genet. 2023 Jan 16;13:1008502. doi: 10.3389/fgene.2022.1008502 (PMC9885372; doi:10.3389/fgene.2022.1008502)

***Fig. 1S***

***
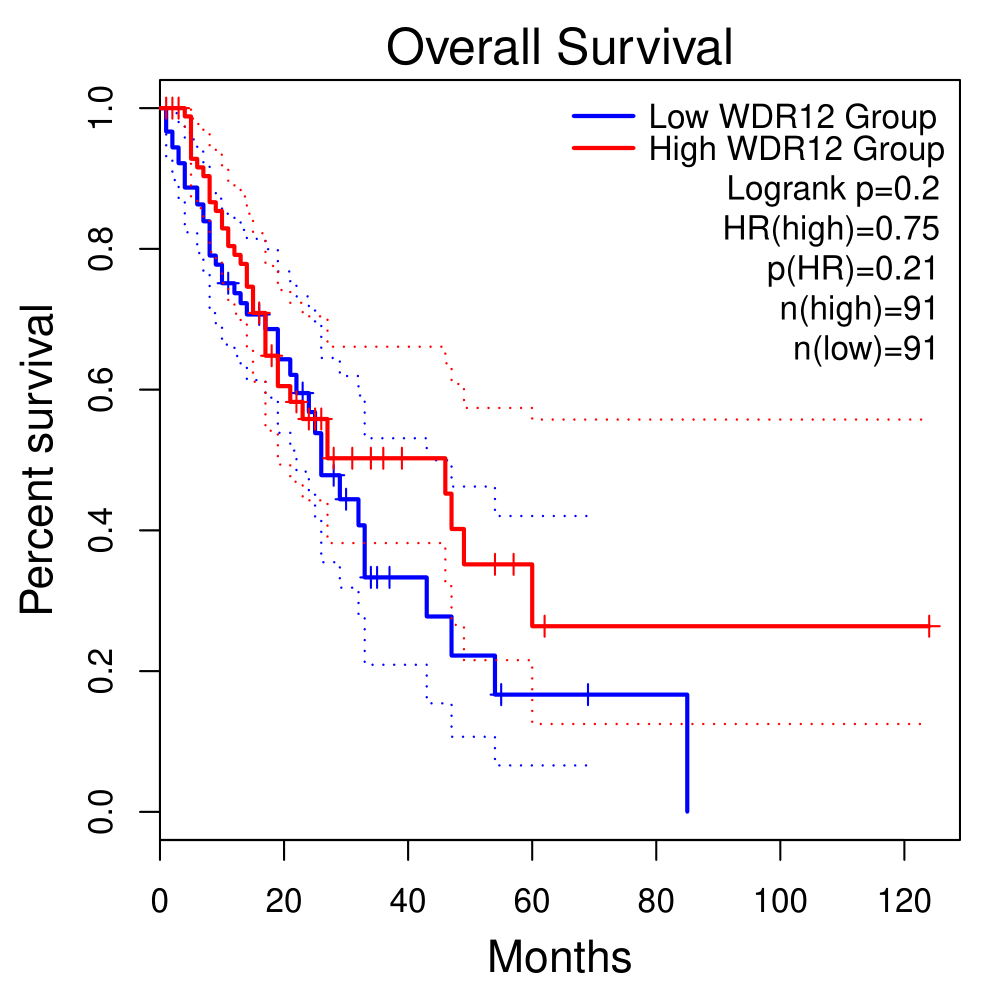

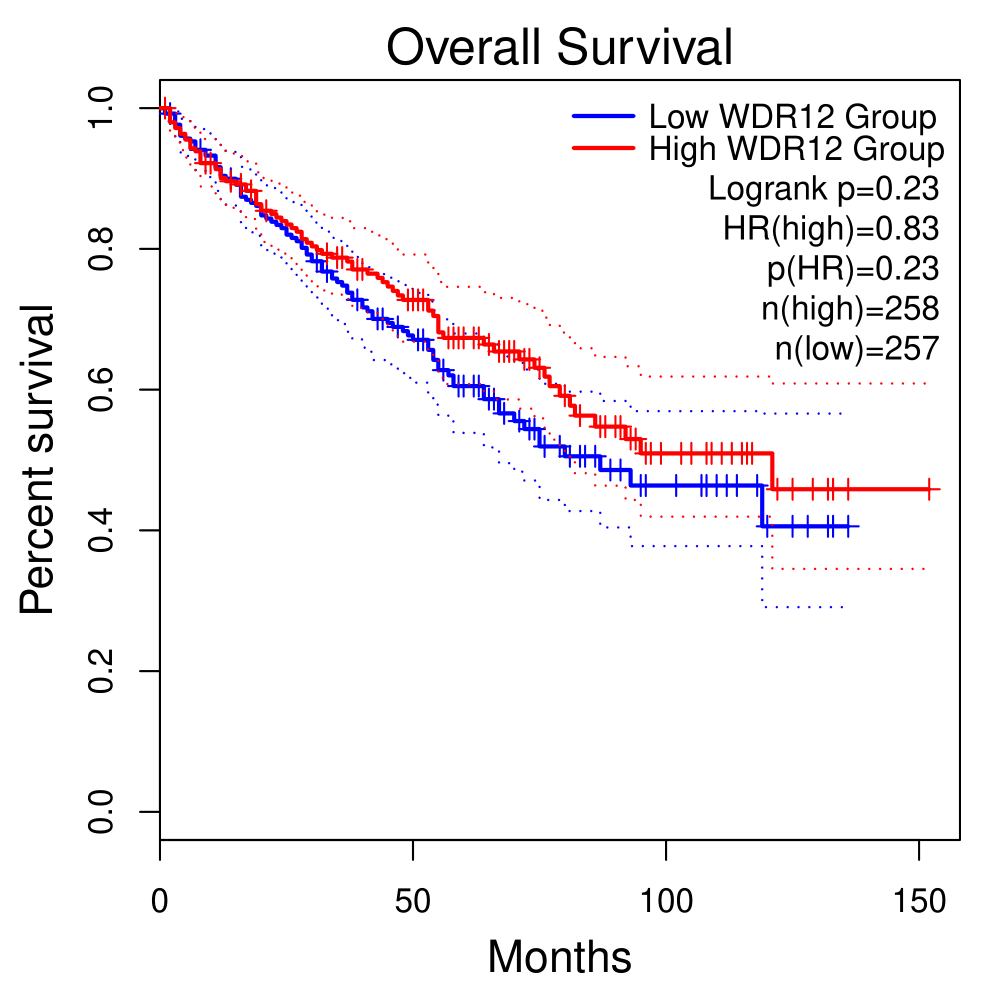

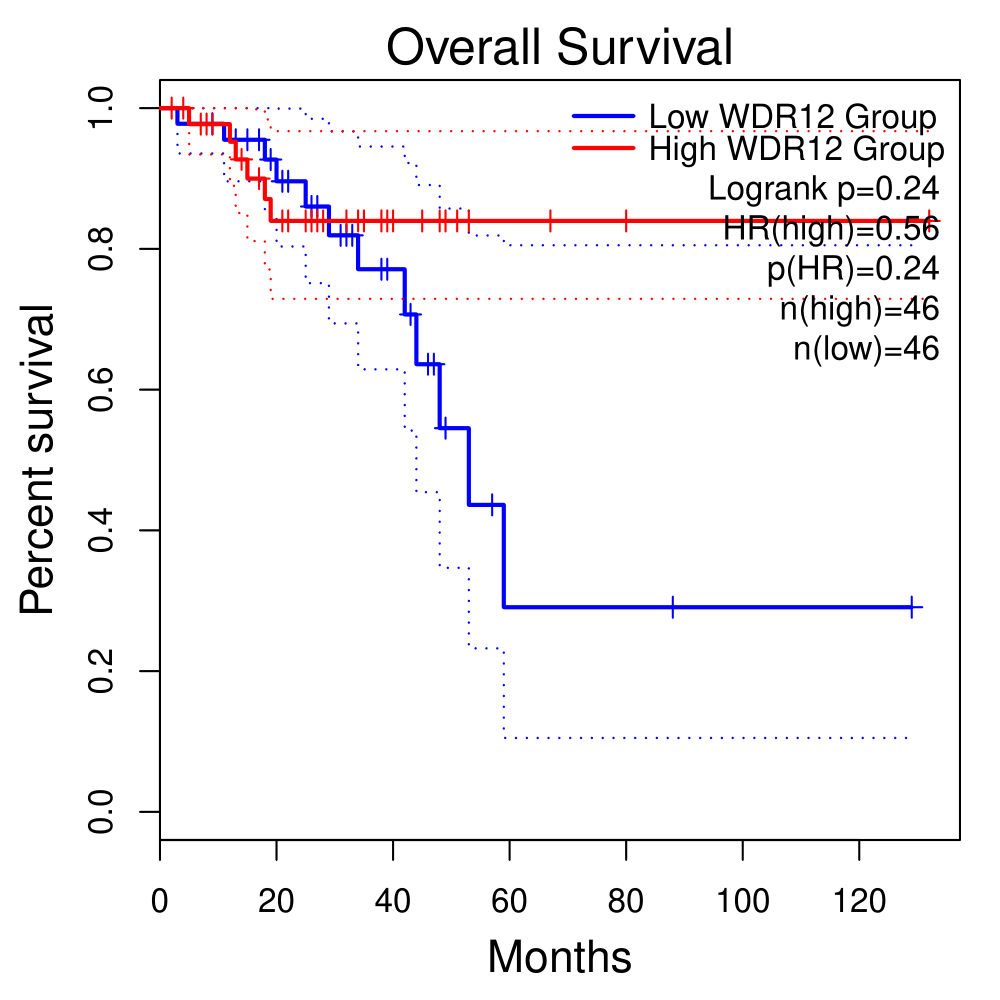

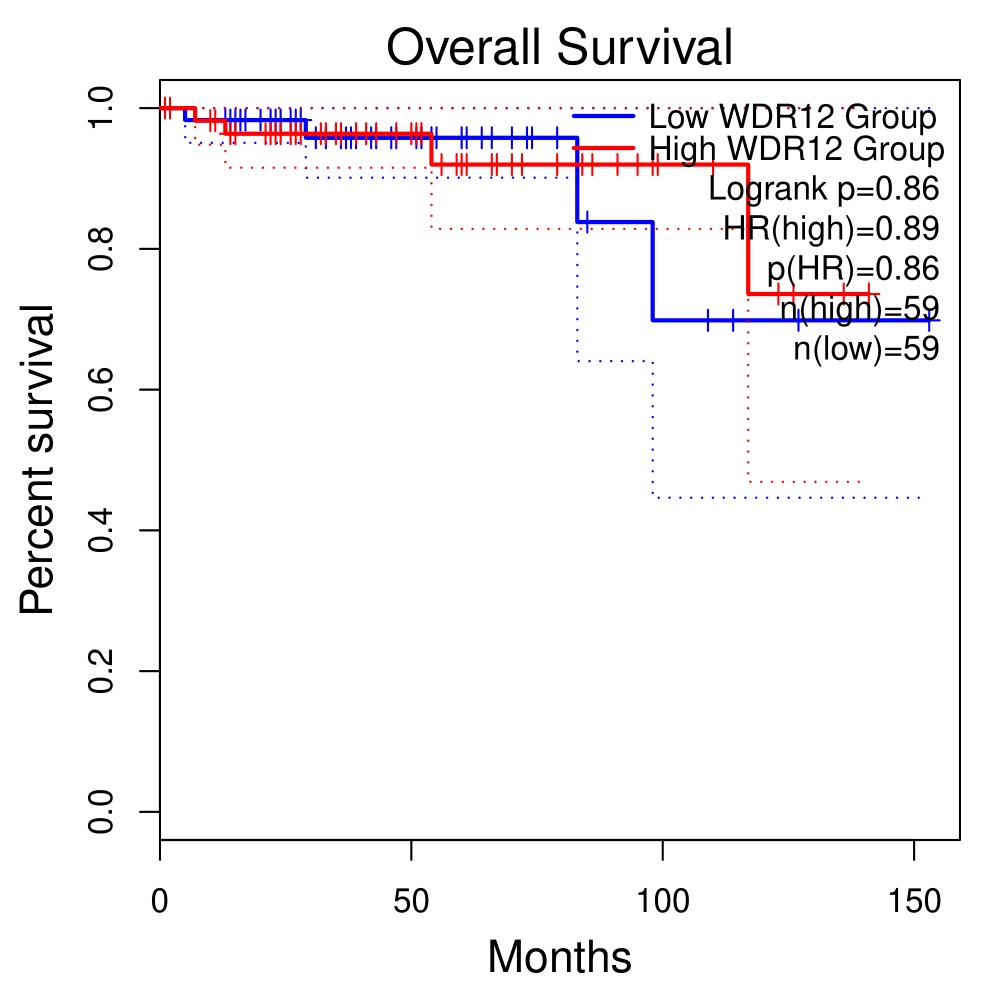
***

***Fig. 2S***


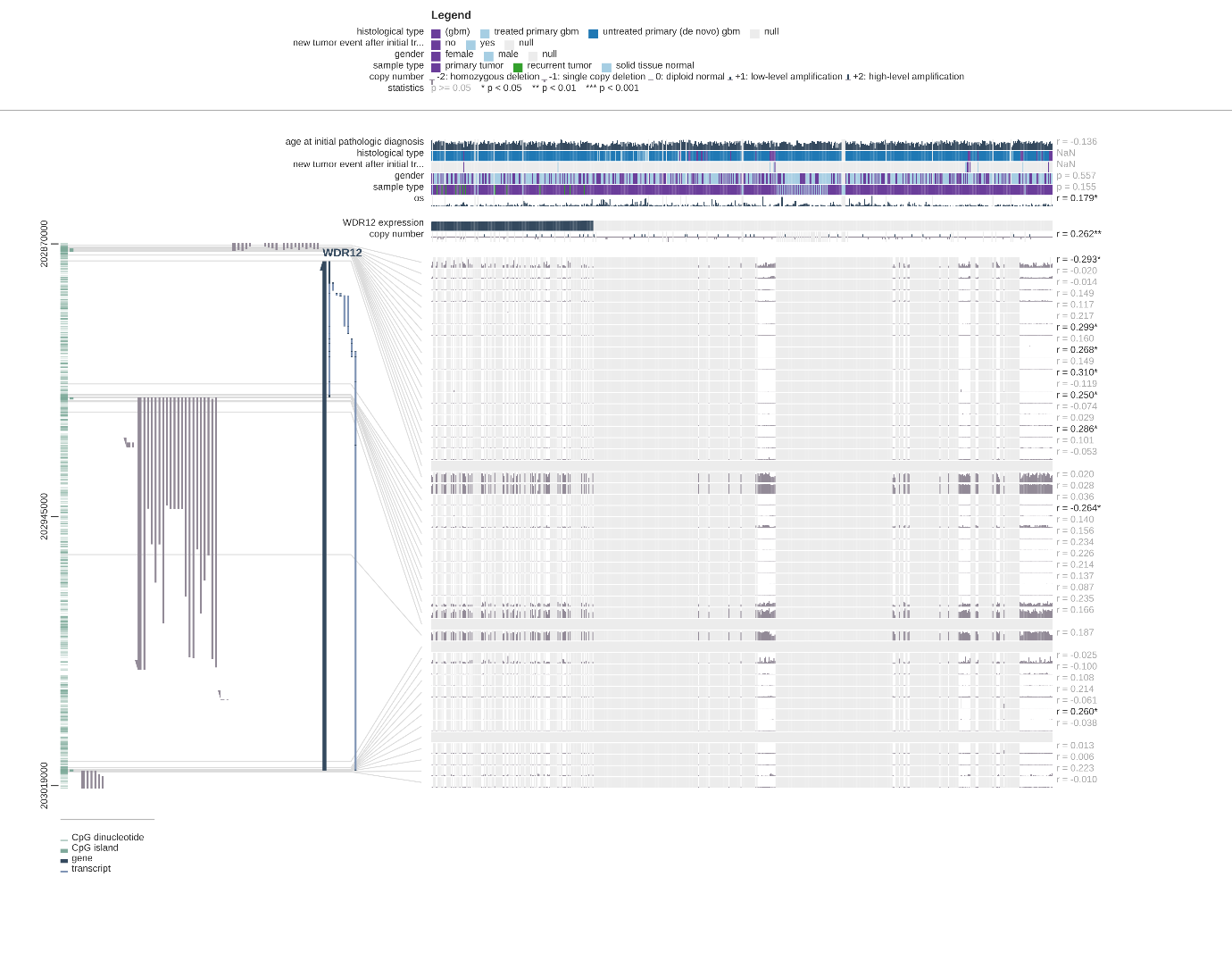

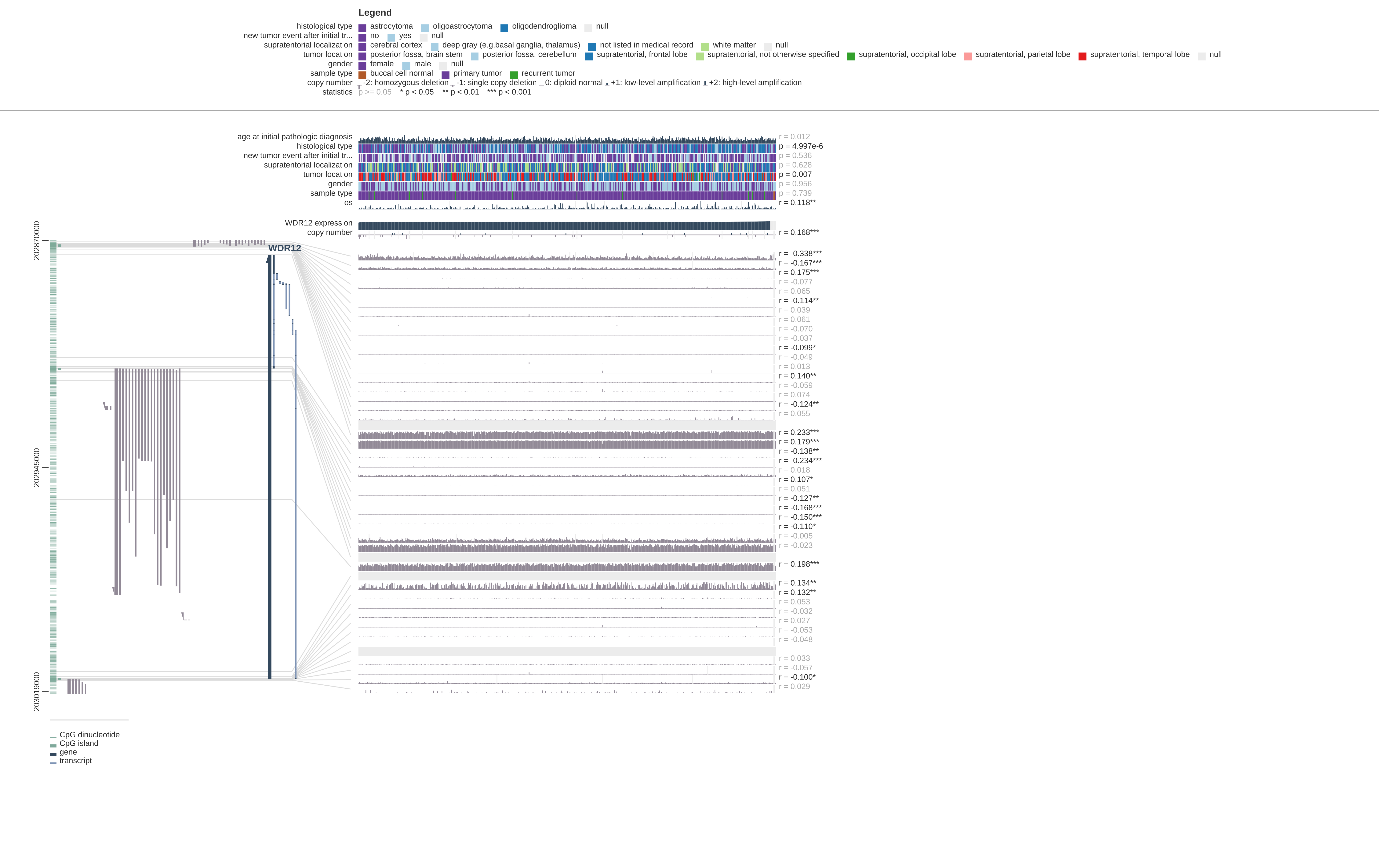


***Fig. S3***


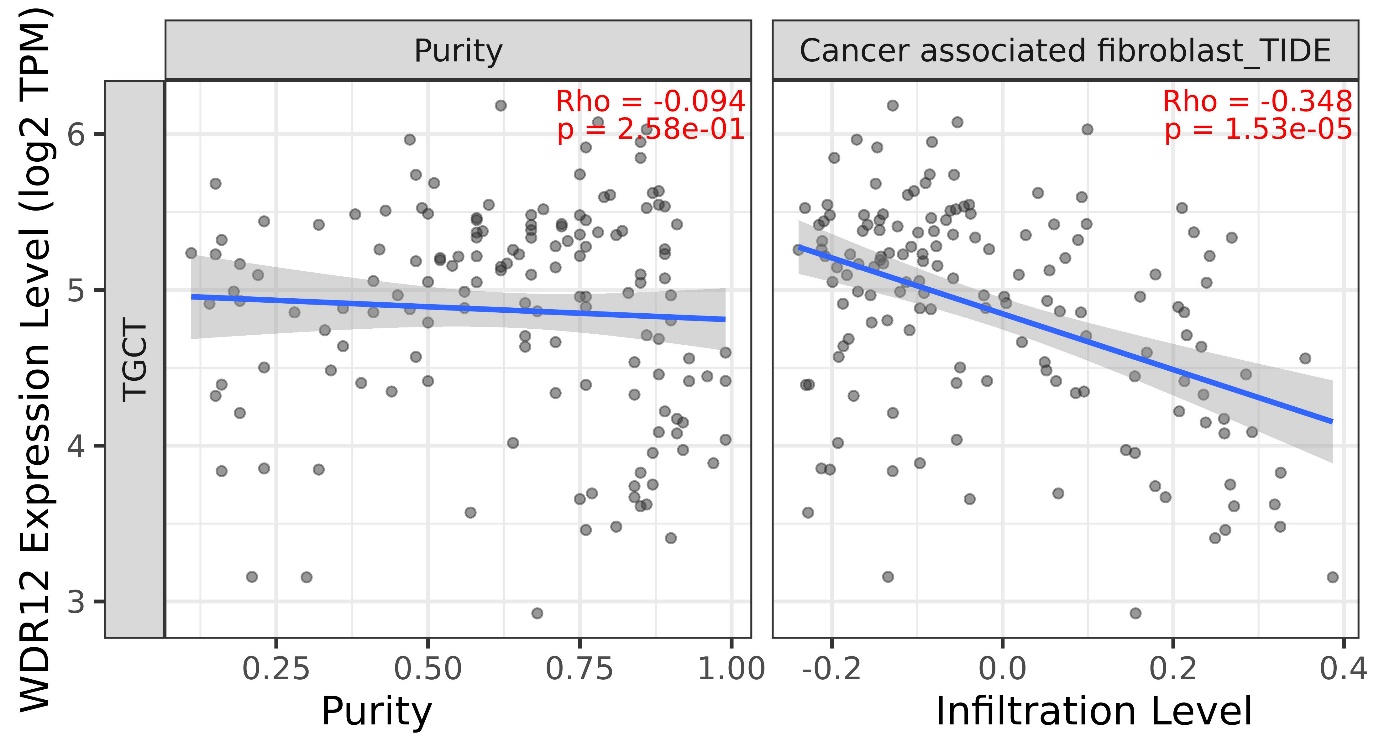

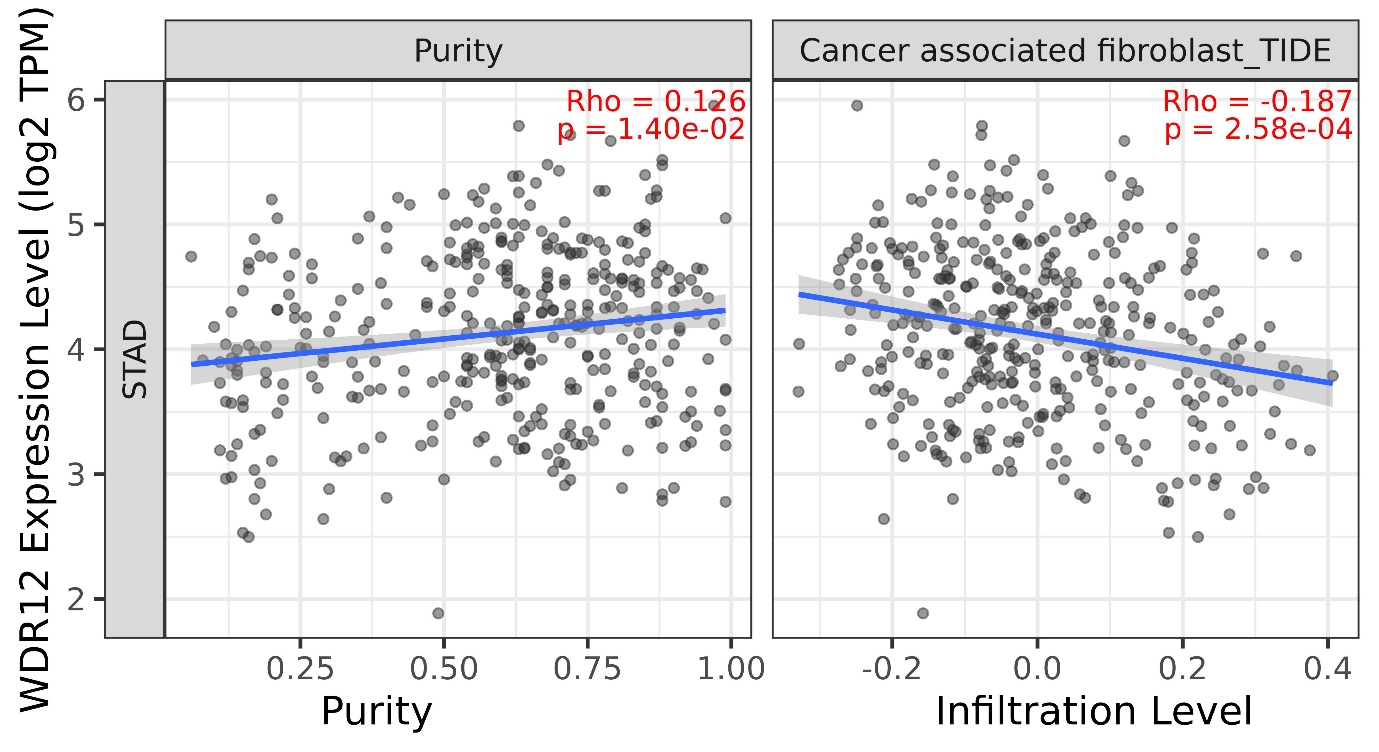

Supplement: Supplementary file 1 [file Table1.DOCX]
